# Genome-Wide Analysis of Coding and Non-coding RNA Reveals a Conserved miR164–NAC–mRNA Regulatory Pathway for Disease Defense in *Populus*

**DOI:** 10.3389/fgene.2021.668940

**Published:** 2021-05-28

**Authors:** Sisi Chen, Jiadong Wu, Yanfeng Zhang, Yiyang Zhao, Weijie Xu, Yue Li, Jianbo Xie

**Affiliations:** ^1^Beijing Advanced Innovation Center for Tree Breeding by Molecular Design, College of Biological Sciences and Technology, Beijing Forestry University, Beijing, China; ^2^National Engineering Laboratory for Tree Breeding, College of Biological Sciences and Technology, Beijing Forestry University, Beijing, China; ^3^Key Laboratory of Genetics and Breeding in Forest Trees and Ornamental Plants, Ministry of Education, College of Biological Sciences and Technology, Beijing Forestry University, Beijing, China

**Keywords:** microRNA, defense response, infection, poplar, *Marssonina brunnea*

## Abstract

MicroRNAs (miRNAs) contribute to plant defense responses by increasing the overall genetic diversity; however, their origins and functional importance in plant defense remain unclear. Here, we employed Illumina sequencing technology to assess how miRNA and messenger RNA (mRNA) populations vary in the Chinese white poplar (*Populus tomentosa*) during a leaf black spot fungus (*Marssonina brunnea*) infection. We sampled RNAs from infective leaves at conidia germinated stage [12 h post-inoculation (hpi)], infective vesicles stage (24 hpi), and intercellular infective hyphae stage (48 hpi), three essential stages associated with plant colonization and biotrophic growth in *M. brunnea* fungi. In total, 8,938 conserved miRNA-target gene pairs and 3,901 *Populus*-specific miRNA-target gene pairs were detected. The result showed that *Populus-*specific miRNAs (66%) were more involved in the regulation of the disease resistance genes. By contrast, conserved miRNAs (>80%) target more whole-genome duplication (WGD)-derived transcription factors (TFs). Among the 1,023 WGD-derived TF pairs, 44.9% TF pairs had only one paralog being targeted by a miRNA that could be due to either gain or loss of a miRNA binding site after the WGD. A conserved hierarchical regulatory network combining promoter analyses and hierarchical clustering approach uncovered a miR164–NAM, ATAF, and CUC (NAC) transcription factor–mRNA regulatory module that has potential in *Marssonina* defense responses. Furthermore, analyses of the locations of miRNA precursor sequences reveal that pseudogenes and transposon contributed a certain proportion (∼30%) of the miRNA origin. Together, these observations provide evolutionary insights into the origin and potential roles of miRNAs in plant defense and functional innovation.

## Introduction

MicroRNAs (miRNAs) are ∼21 to 24-nucleotide (nt) non-coding endogenous small RNAs that can regulate gene expression, maintain genome integrity and chromatin structure, and influence plant development and stress response (Carrington and Ambros, 2003; Jones-Rhoades et al., 2006; Voinnet, 2009; Sunkar et al., 2012; Meyers and Axtell, 2019). Under pathogen stress, basal defense and resistance gene-mediated resistance are the two well-defined defense responses carried out by plants. Innate immunity is an evolutionarily ancient mechanism that protects plants from a wide range of pathogens (Peláez and Sanchez, 2013). Many lines of evidence have confirmed that miRNAs contribute to plant defenses against pathogens (Li et al., 2012; Pumplin and Voinnet, 2013; Thiebaut et al., 2015; Yang and Huang, 2015). Evolutionary analyses revealed that a miRNA superfamily composed of the miR482 and miR2118 families targets the plant nucleotide-binding leucine-rich-repeat (NB-LRR) defense genes (Zhao et al., 2015). Moreover, miRNAs–transcription factor (TFs) regulation module was proposed to be ubiquitous in plant defense and plays key roles in regulation networks controlling many biological processes, including responses to biotic and abiotic stresses (Seo et al., 2015; Thiebaut et al., 2015).

As we know, the *Populus* genus consists of many important woody species, such as the western balsam poplar (*Populus trichocarpa*) (Tuskan et al., 2006), the desert poplar (*Populus euphratica*) (Ma et al., 2013), and the Chinese white poplar (*Populus tomentosa*) (Du et al., 2014). Several species have been selected as model tree species for their small genome size and rapid growth. The availability of reference genome sequences for *Populus* species thus makes them important model systems for the investigation of miRNA functions during pathogen infections. To date, hundreds of miRNAs have been identified in *Populus*, and the function of several well-known miRNAs has been clarified in literatures (Zhang et al., 2010; Kozomara and Griffiths-Jones, 2014). During the infection of bacterial or fungal pathogens, the transcription patterns of poplar miRNAs were highly associated with the disease resistance (DR) response (Zhao et al., 2012; Li et al., 2016). An economically important group of poplar pathogens, *Marssonina brunnea*, is a typical hemibiotrophic fungal pathogen, which can cause disease *Marssonina* leaf spot of poplars (MLSP) (Zhang et al., 2018). Although MLSP has been studied for over 30 years, the key non-coding RNAs that function during *M. brunnea* infection and their effects on plant defense are poorly understood. Therefore, increasing molecular understanding of the plant-*M. brunnea* interaction will be helpful for the development of control strategies against MLSP.

In the present study, we combined transcriptome and genomic analyses to explore the origin, evolution, functional innovation, and plant defense effects of the poplar miRNAs during the three essential stages of MLSP fungus (*M. brunnea*) infection, including conidia germinated stage [12 h post-inoculation (hpi)], infective vesicle stage (24 hpi), and intercellular infective hyphae stage (48 hpi), as described in Chen et al. (2020). By exploring the origin and evolutionary patterns of poplar miRNAs, our study provides new insight into the feedback regulation mechanism of miRNAs. Besides, our study attempts to compare the regulation mechanism between conserved and *Populus*-specific miRNA to achieve a better understanding of the coevolution between miRNAs and target genes. Finally, a conserved hierarchical regulatory network combining promoter analyses and hierarchical clustering approach reveals a miR164–NAM, ATAF, and CUC (NAC) transcription factor–messenger RNA (mRNA) regulatory module that has potential in *Marssonina* defense responses. Overall, this study reveals the evolutionary patterns and illustrates the functional novelty of lineage-specific miRNAs in DR processes.

## Materials and Methods

### Plant Materials and Fungal Treatments

The Chinese white poplar (*P. tomentosa* cv. “LM50” clones) was planted in pots under natural light conditions (12 h of 1,250 μmol m^–2^ s^–1^ photosynthetically active radiation) at 25°C ± 2°C (day and night) and 50% ± 1% relative humidity (day and night) in an air-conditioned glasshouse using soilless culture technology. The infection experiments were performed using *M. brunnea* f. sp. *Monogermtubi* strain bj01. Six inoculation spots per poplar leaf were each inoculated with 5 μl of 10^5^ condia ml^–1^, with three biological replicates per treatment. The spore suspension was sprayed onto the abaxial surfaces of the leaves *in vitro*, and the inoculated leaves were incubated in an artificial climate incubator (LT36VL, Percival Scientific, Inc., Perry, United States) under 25°C and 95% relative humidity and harvested at 12, 24, and 48 hpi. In the control group, the leaves were sprayed with sterile tap water and harvested at 12 hpi. The samples from leaves exposed to the same treatment were pooled and treated as one biological repeat, and two independent experimental repeats were performed for each treatment (CK, 12, 24, and 48 hpi). All samples were immediately frozen in liquid nitrogen and stored at –80°C for RNA extraction.

### MicroRNA Library Construction

Total RNA was extracted from inoculated leaf samples using a TRIzol reagent (Invitrogen, Carlsbad, CA, United States) according to the manufacturer’s instructions. Additional on-column DNase digestion was performed during the RNA purification using RNase-Free DNase (Qiagen). The total RNAs were ligated with 3′ and 5′ adapters using a Small RNA Sample Prep Kit (Illumina). sRNAs with adapters on both ends were used as templates to create cDNA constructs using reverse transcription PCR. After being purified and quantified using a Qubit dsDNA HS (Qubit 2.0 Fluorometer) and Agilent 2100, the PCR products were used for cluster generation and sequencing on an Illumina HiSeq 4000 according to the cBot and Hiseq 4000 user guides, respectively.

### Messenger RNA Sequencing, Alignment, and Normalization

mRNA reads were aligned using TopHat2 (Kim et al., 2013) using*—read-mismatches* 2*-p-G* to generate read alignments for each sample. Up to two mismatches were permitted in each read alignment. The transcript abundance was calculated, and differential transcript expression was computed using CuffDiff with the parameters*-p-b* (Ghosh and Chan, 2016).

### Small RNAome Analysis

The sequences generated from the leaves exposed to the three infection treatments were used to detect the transcript abundance of mature sRNAs. All sRNA reads, referred to as raw reads, were processed to remove adaptors, low-quality tags, and contaminants. Clean reads were then mapped to version 3.0 of *P. trichocarpa* genome with no more than one mismatch. These perfectly aligned sequences were annotated by BLAST-searching them against the GenBank and Rfam databases (version 13^1^), allowing one mismatch. The tRNAs, rRNAs, snRNAs, snoRNAs, and scRNAs were removed from the sequencing reads. The remaining unannotated sRNAs were searched against the known miRNAs from miRBase version 22.1^2^, allowing a maximum of two mismatches. Then, the remaining unannotated unique sequences were mapped to the *P. trichocarpa* genome to uncover novel miRNAs from poplar, according to the established criteria (Meyers et al., 2008), using Mireap software^3^. Finally, only miRNAs with high expression levels (actual count of reads exceed 10 in at least one sample) and loci that could produce both mature miRNAs and antisense miRNA (miRNA^∗^) sequences were kept in our study.

### Genomic Locations of MicroRNAs in the ***P. trichocarpa*** Genome

The pre-miRNAs were screened for their localization within the transposons, introns, exons, pseudogenes, intergenic regions, 5′ untranslated region (UTR), 3′ UTR, coding sequence (CDS), and promoter (upstream 2 kb of coding genes) region of the *P. trichocarpa* genome v3.0 (Phytozome version 12), with an overlapping rate of above 80%.

### Target Prediction and Functional Annotation

Targets of each miRNA were predicted using Web server psRNATarget^4^, with *P. trichocarpa* transcript (phytozome v10.0, genome V3.0, internal number 210) as target gene search scope, the expectation is less than 5, and other parameters as default. The functional annotation and categorization of candidate miRNA targets were performed using the AgriGO software suite v2.0^5^ with default parameter (Tian et al., 2017). Regulatory networks were drawn using Cytoscape version 3.8.0 (Shannon, 2003).

### Transposon Element Annotation

The transposon element (TE) annotations used in this study were obtained from the outputs of the RepeatMasker (RM) software version 4.0.7 combined with the database (Dfam_Consensus-20170127, RepBase-20170127; -species parameter: *Populus*). These RM outputs were filtered to remove non-TE elements, such as satellites, simple repeats, low complexity sequences, and rRNA.

### Pseudogene Annotation

The intergenic sequences of the *P. trichocarpa* genome were used to identify the putative pseudogenes. The overall pipeline used for this identification was generally based on the PlantPseudo workflow (Xie et al., 2019) and consisted of four major steps: (1) identify the masked intergenic regions with sequence similarity to known proteins using BLAST; (2) eliminate redundant and overlapping BLAST hits in places where a given chromosomal segment has multiple hits; (3) link homologous segments into contigs; and (4) realign sequences using tfasty to identify features that disrupt contiguous protein sequences.

### Real-Time Quantitative PCR

RT-qPCR was performed on a 7,500 Fast Real-Time PCR System (Applied Biosystems, Waltham, MA, United States) using the SYBR Green *Premix Ex* Taq II (TaKaRa). All primer pairs for the candidate genes were designed by an online tool provided by Integrated DNA Technologies^6^, as shown in Supplementary Data 1. Poplar 18S rRNA was used as an internal control for gene expression measurements for target genes. The Mir-X^TM^ miRNA qRT-PCR SYBR Kit (Clontech, Mountain View, CA) was used. The relative expression level of each miRNA was measured and standardized to 5.8S rRNA. The relative expression of miRNAs and target genes was calculated using the 2^–△△Ct^ method.

## Results

### Expression Dynamics of Poplar MicroRNAs During the *Marssonina* Infection

**FIGURE 1:**
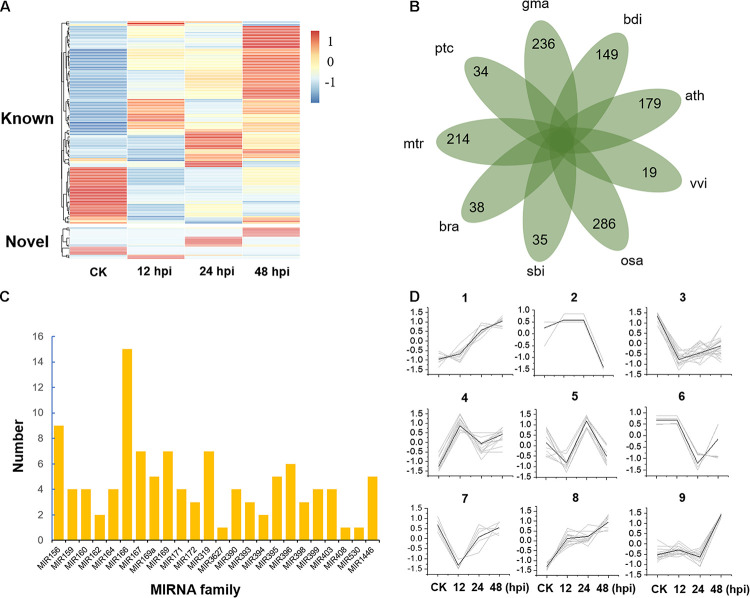
Expression and classification of microRNAs (miRNAs). **(A)** Expression of known and novel miRNAs. hpi, hours post-inoculation. **(B)** The species-specific miRNA families of nine plant species. ptc, *Populus trichocarpa*; mtr, *Medicago truncatula*; bra, *Brassica rapa*; sbi, *Sorghum bicolor*; osa, *Oryza sativa*; vvi, *Vitis vinifera*; ath, *Arabidopsis thaliana*; bdi, *Brachypodium distachyon*; gma, *Glycine max*. **(C)** The distribution of 110 conserved miRNA members in 23 miRNA families. **(D)** Nine clusters were obtained by *K-*means clustering with Euclidean distance as the distance metric.

To study the posttranscriptional regulation associated with poplar defense to *Marssonina*, we inoculated the leaves of *P. tomentosa* LM50 clones with *M. brunnea* f. sp. *Monogermtubi* bj01 conidial suspension, and the expression pattern of miRNAs was investigated by small RNA sequencing. Specifically, we prepared a library of RNAs of 18–30 nucleotides (nt) from each sample (CK, 12, 24, and 48, two biological repeats), generating 17.5 million reads in total; 83–86.7% of the total reads could be aligned perfectly (no more than one mismatch) to the *P. trichocarpa* genome (version 3.0) (Supplementary Data 2; Tuskan et al., 2006). In total, we identified 131 miRNA precursors by alignment to miRbase v22.1, which were grouped into 37 miRNA families containing an average of 3.5 genes per family (Supplementary Data 3). A total of 21 sequences were identified as potential novel miRNAs, with average minimum free energy (MFE) of -58.4 kal/mol (Supplementary Figure 1 and Supplementary Data 3). Compared to known miRNAs, the expression of novel miRNAs was generally low (Figure 1A and Supplementary Data 4). Some novel miRNAs were discovered in only one of the libraries partly because the sequencing depth provided insufficient coverage of all the miRNAs or some miRNA expressions are specifically turned on or turned off by pathogen stress. A total of 110 conserved miRNAs belonging to 23 miRNA families and 42 *Populus*-specific miRNAs belonging to 34 miRNA families were identified (Figures 1B,C and Supplementary Data 5).

To study the global expression patterns, we also performed k-means clustering to describe the expression of miRNA during poplar response to *M. brunnea*. We detected nine co-expression clusters (Figure 1D and Supplementary Data 6). Cluster 1 and cluster 8 contained 18 and 25 miRNAs, respectively, showing a consistent increase during defense response. While cluster 3 showed a reverse trend. Cluster 9 contained 21 miRNAs, which show their expression peaks at 48 hpi. MiRNAs of cluster 4 and cluster 5 showed their expression peaks at 12 and 24 hpi, respectively (Figure 1D and Supplementary Data 6). Gene Ontology (GO) enrichment analysis showed that most of the target genes of cluster 1 and cluster 8 are involved in cell adenyl ribonucleotide binding, ATP binding, and protein kinase activity. Targets from miRNAs in Cluster 3 were significantly enriched in the biological process of regulating the primary metabolism, biosynthesis, and transcription (*P* < 1 × 10^–4^). Overall, miRNAs play essential roles in stress response by activating or suppressing the expression of their target genes.

### Pseudogenes and Transposons Act as Catalysts for the Formation of MicroRNA

**FIGURE 2:**
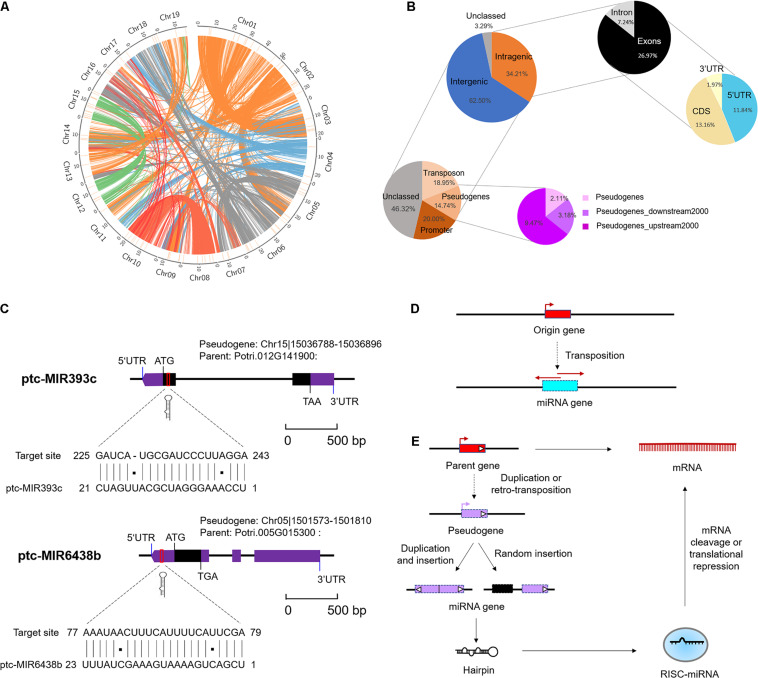
Genomic locations of microRNAs (miRNAs) in *Populus trichocarpa* genome. **(A)** Genomic distribution of 152 *Marssonina brunnea* responsive miRNAs. Chromosomes are represented by the circle, and the inner circles (short orange lines) represent the location of miRNAs at the genome. The central colorful lines represent lines that connect syntenic block across chromosomes. **(B)** miRNA locations at the intergenic and protein-encoding genes (PEGs) region. The two charts on the top right indicate the locations of miRNA precursor sequences (*MIRs*) in the regions of PEGs. The two charts on the bottom left indicate the detailed classifications of *MIRs* overlapping with transposon, pseudogenes, and promoter region. **(C)** miRNA origin from pseudogenes and targeted parent gene. miR393c and miR6438b were selected as representative miRNAs. **(D)** Mechanism of miRNA gene origin from transposon. **(E)** Mechanism of miRNA gene origin from pseudogenes and feedback regulated the parent gene.

To elucidate the underlying mechanism of emergence of (conserved and *Populus*-specific) miRNAs, we examined the locations of miRNA precursor sequences (*MIRs*) in the regions of *P. trichocarpa* genome, including intragenic regions (exons, introns, CDS, and UTRs) and intergenic regions (Supplementary Data 7). These conserved or *Populus*-specific miRNAs were extensively distributed in poplar genomes (Figure 2A). Of these miRNAs, 52 (34.21%) were located within protein-encoding genes (PEGs), five (3.29%) were in unclassified sequences (scaffold), and 95 (62.50%) were in the intergenic region (Figure 2B). Of the 52 miRNAs within PEGs, 11 (7.24%) were in intron regions, 20 (13.16%) were in CDS regions, 18 (11.84%) were in 5′ UTR regions, three (1.97%) were in 3′ UTR regions (Figure 2B). Notably, nearly half of these intragenic-derived miRNAs (24 out of 52) showed the same transcriptional orientation as their host genes, indicating that the transcription of the miRNAs may associate with the host genes.

Moreover, we further examined the location of *MIRNA* precursors in relation to transposon region, pseudogenes, and promoter region (2-kb sequences of genes upstream) in poplar genome. As a result, we detected 24 (15.79%) miRNAs in the transposon region, 22 (14.47%) were in pseudogenes, and 23 (15.13%) were in the promoter region (Figure 2B and Supplementary Data 7). Notably, the proportions of miRNAs located in pseudogenes and transposons were significantly larger than expected by chance (*P* < 1 × 10^–3^, one-sided z test). This suggests that pseudogenes and TEs may contribute to the origin of *Populus* miRNAs. A careful examination of their precursor sequences revealed that *Populus*-specific miR478e and miR6427 were transposons-derived miRNAs; conserved miR393c and *Populus*-specific miR6438b were pseudogenes-derived miRNAs (Figure 2C). The *Populus*-specific miR6438b was derived from upstream 192–215 bp of pseudogene Chr13| 15188492-15190233 and targeted the 5′ UTR of its parent gene (Potri.005G015300); and conserved miR393c were derived from 2 to 23 bp of pseudogene Chr04| 22273397-22273670 and targeted the CDS of its parent gene (Potri.012G141900) (Figure 2C). Together, the strong association of miRNAs with pseudogenes and TE provides an important mechanism for the origin and posttranscriptional regulation of miRNAs (Figures 2D,E).

### The *Populus* MicroRNAs Fine-Tune the Expression of Disease Resistance Genes

**FIGURE 3:**
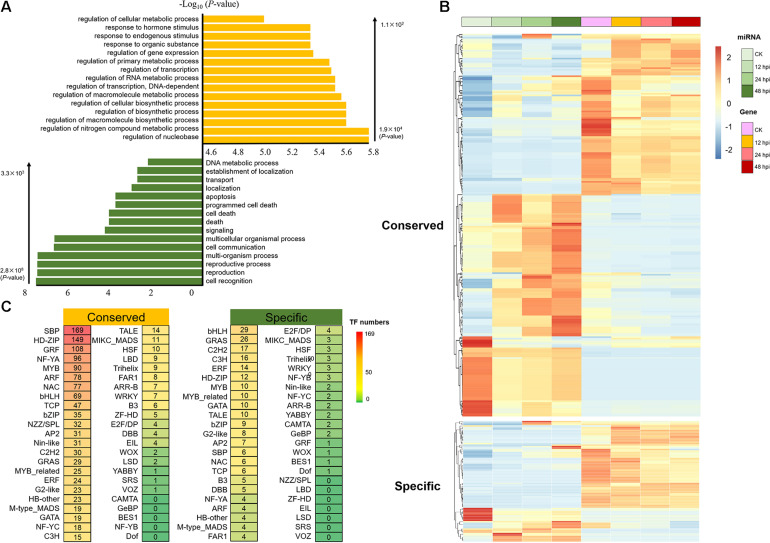
Analysis of the target of conserved and *Populus*-specific miRNAs. **(A)** Significantly enriched Gene Ontology biological processes for target genes of conserved and *Populus*-specific miRNAs, respectively (Top 15; *P* < 1 × 10^– 2^). **(B)** Expression of the miRNA/target genes with negatively correlated expression patterns (*r* < –9 × 10^– 1^, *P* < 5 × 10^– 2^; Pearson correlation). **(C)** The number of transcription factor (TF) targets of conserved and *Populus*-specific miRNAs, respectively.

To investigate the regulatory networks associated with these *M. brunnea*-responsive miRNAs, 12,839 predicted miRNA-target gene pairs were identified by using psRNATarget (Dai and Zhao, 2011). It contains 8,938 conserved miRNA-target gene pairs and 3,901 *Populus*-specific miRNA-target gene pairs (Supplementary Data 8). The results of GO functional enrichment showed that target genes of conserved miRNA were mainly concentrated on regulating metabolism and transcription, whereas *Populus*-specific miRNAs were significantly enriched in the process of signaling and programmed cell death (*P* < 1 × 10^–2^; Figure 3A). An examination of the expression of the miRNA/target genes revealed 321 pairs with negatively correlated expression patterns (r < –9 × 10^–1^, *P* < 5 × 10^–2^; Pearson correlation; Figure 3B and Supplementary Data 9). Using the publicly available degradome library (Xie et al., 2017), we identified 247 miRNA/target pairs (Supplementary Data 10), of which several conserved miRNA/target pairs (miR156-*SPL*, miR164-*NAC*, and miR172-*RAP*) were observed in our dataset (Wang et al., 2009, 2015, 2020).

**FIGURE 4:**
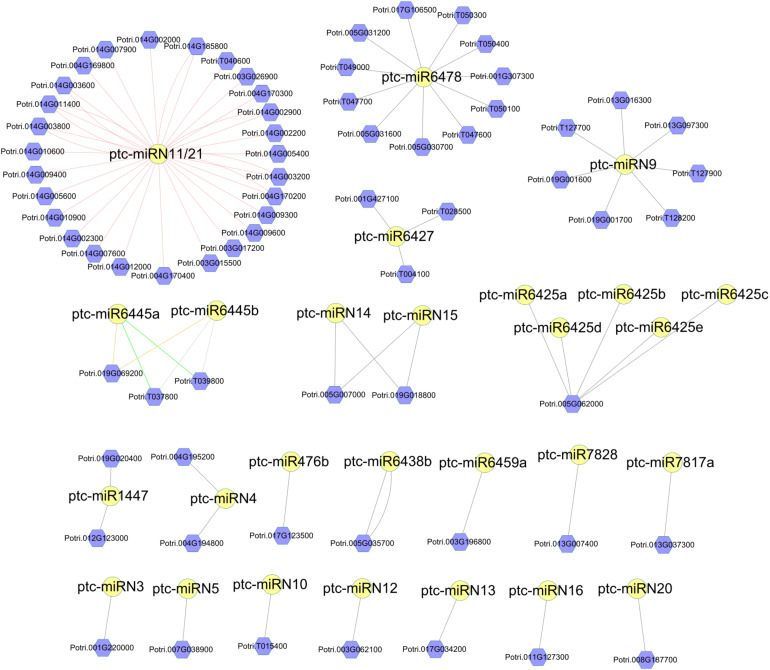
Network of *Populus*-specific miRNA/disease resistance (DR) target gene pairs. The nodes with yellow circles are miRNAs. The nodes with violet hexagon are DR genes. The lines between miRNAs and DR genes represent the targeting relationship.

Next, we performed target prediction analyses. As a result, a total of 114 DR genes and 123 DR genes were predicted to be the targets of the conserved and *Populus*-specific miRNAs, respectively. Notably, a larger proportion of *Populus*-specific miRNAs (28 of 42) was found to target DR genes than that of conserved miRNAs (60 of 110). The results suggest that *Populus-*specific miRNAs were more involved in the regulation of the DR genes (*P* = 1.07 × 10^–11^; Fisher’s exact test; Supplementary Data 8). For instance, a 22-nt *Populus*-specific ptc-miRN11 and a 23-nt *Populus*-specific ptc-miR6478 were predicted to target 35 and 11 DR genes, respectively (Figure 4). To gain a better understanding of the functional roles of miRNAs, we next performed a pfam domain analysis of DR targets. A total of 48 TIR-NBS-LRR (TNL), 19 CC-NBS-LRR (CNL), 99 NBS (N), 61 NBS-LRR (NL), one TIR, and two LRR family proteins were detected. Among them, two 24-nt *Populus*-specific families, ptc*-*miR6445 and ptc*-*miR1447, were predicted to target a TNL (Potri.019G069200) and an N (Potri.012G123000) DR gene, respectively. Notably, an examination of the expression patterns of the DR genes showed that these genes were expressed at a very low level at all-time points. Thus, these results indicated that susceptibility genotype increased the resistance partly by fine-tuning the DR genes.

### The Conserved MicroRNA–Transcription Factor Model Supports the Gene Dosage Balance Hypothesis

To explore the role of miRNA–TF pairs in fungi pathogen-stress response, we next analyzed the posttranscriptional regulation of 152 miRNAs. A total of 1,603 miRNA–TF pairs were detected, including 1,384 conserved miRNA–TF pairs and 219 *Populus-*specific miRNA–TF pairs (Supplementary Data 11). Here, we observed that miRNAs target numerous TFs that associated with defense response during the *M. brunnea* infection, such as *SBP*, *NAC*, *NF-YA*, *ARF*, and *MYB* (Figure 3C). SBP is a well-known TF family in plants, which is involved in various stress response networks (Liu et al., 2019). In total, 152 conserved miRNA–*SBP* regulation pairs were detected; 83 miRNA–NAC regulation pairs (78 conserved and five *Populus*-specific) were detected, for example, *NAC1* and *NAC100* (*NAC1*: Potri.007G065400; *NAC100*: Potri.012G001400) were negatively correlated with miR164a-d; 82 miRNA–ARF regulation pairs were detected; 110 miRNA-MYB regulation pairs were detected. Thus, this implied that miRNAs might play important roles in regulating a wide range of molecular events during the *M. brunnea* infection.

To study the evolutionary effects of polyploidy on a transcriptional network, we reanalyzed the functional genomic and transcriptome data for numerous duplicated gene pairs formed by ancient polyploidy events in poplar (Rodgers-Melnick et al., 2012). A total of 5,931 “salicoid duplications” targeted by miRNAs were detected, including 1,023 WGD-derived TF pairs (Supplementary Datas 12, 13). Notably, of these TF pairs, 459 (∼44.9%) have only one paralog being targeted by a miRNA. This could be due to either gain or loss of the miRNA binding sites of one of the duplicates after WGD. Furthermore, detailed analysis revealed that 83.7% of the TF WGDs were targeted by conserved miRNAs. This could be explained by gene balance hypothesis. Under the hypothesis, we expected that more conserved miRNAs would target genes of central roles in networks such as functional TFs. Overall, miRNAs play important roles in biological regulating network, with conserved miRNAs regulating central biological nodes, supporting the gene balance hypothesis.

### MiR164–NAC–mRNA Regulatory Network in Response to Biotic Stress in *Populus*

To further study the complexity of the transcription regulatory network in response to biotic stress, we carried out motif occurrence analysis of five groups of disease-resistant related genes, including signaling cascades, TFs, reactive oxygen, pathogen-related, and NBS. As a result, 52,164 TF binding sites were enriched in the promoters of detected genes with a frequency that exceeds 85% (Supplementary Data 14). Many DR-related genes were predicted to be the upstream TFs in our data set, such as NAC, MYB, WRKY, ERF, and bZIP. In particular, plant NAC domain protein may serve as a convergent node in developmental processes and stress response. For instance, NAC was found to increase necrotrophic/biotrophic pathogen tolerance, which could be induced by wounding and defense-related hormones (Galle et al., 2013). Also, overexpression of NAC4 (ANAC079/080) in *Arabidopsis* could increase the pathogen stress tolerance (Lee et al., 2017).

**FIGURE 5:**
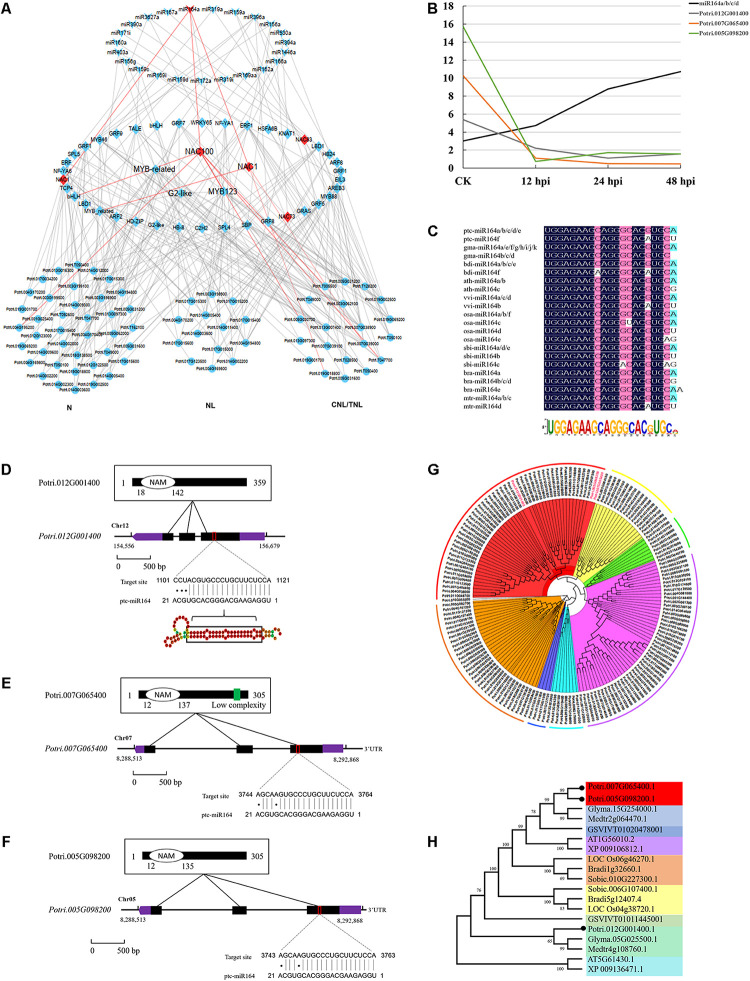
Conserved miR164–NAC–mRNA regulatory network in response to fungi pathogen stress in *Populus*. **(A)** The three-layered gene regulatory network (GRN) was constructed with the backward elimination random forest (BWERF) algorithm. The nodes with red color highlighted the key regulatory transcription factors (TFs). **(B)** The expression value of miR164 and *NAC1/100* genes. **(C)** Sequence logo view of the mature miR164 sequence. **(D–F)** Conserved domains of ptc-NAC1/100 protein sequence, gene structure of *ptc-NAC1/100*, and predicted base-pairing interaction between ptc-miR164 and *ptc-NAC1/100*. Exons are shown as *black boxes* and introns as *lines*. The 5′ UTR and 3′ UTR are shown as *purple boxes.* **(G,H)** Phylogenetic analysis of *NAC* targets of miR164 in *Populus*. Phylogenetic analysis of *ptc-NAC1/NAC100* homologous genes in eight other plant species.

To further study the regulatory network of miRNAs involved in DR, we next performed hierarchical clustering analysis on TFs (targets of conserved miRNAs) and DR genes. We thus constructed a three-layer network uncovering the module of miR164–*NAC*–mRNA, with an important role in the fungal pathogen infection (Figure 5A). This module includes three *NAC* genes (*NAC1*: Potri.007G065400; *NAC100*: Potri.012G001400; *NAC1*: Potri.005G098200) whose expressions were negatively correlated with miRNA164a (Figures 5A,B). This was also supported by the real-time quantitative PCR analyses (*P* < 0.05; Supplementary Figure 2). Sequence conservation analyses showed that the mature regions of miR164a were completely conserved in *Arabidopsis*, rice, maize, *Medicago*, *Brassica*, *Sorghum*, *Vitis*, *Brachypodium*, and *Glycine*, and the precursor sequences of miR164a show an extensive similarity (41.76%) in eight plants (Figure 5C). Moreover, analysis of the genomic and protein sequences of the three *NAC* genes showed that all of them were composed of three exons and two introns and evolutionarily conserved (Figures 5D–H). In total, 134 genes were predicted to be the downstream targets of the three NAC (Supplementary Data 14). Functional enrichment analysis showed that these genes were mainly involved in biological processes such as apoptosis and innate immune response (*P* < 1e-56). Taken together, a multilayered hierarchical gene regulation network provides opportunities to investigate transcriptome dynamics and identifies key genes involved in specific pathways.

## Discussion

### Pseudogenes and Posttranscriptional Regulation

The origins of miRNA genes have attracted wide attention in recent years. In plants, there are at least four hypotheses, for instance, according to sequence homology between *MIR* genes and target genes, Allen et al. (2004) proposed the inverted duplication hypothesis. Under the hypothesis, these young miRNA genes were supposedly generated from inverted duplication events of one of their target genes by forming two adjacent gene segments in either convergent or divergent orientation. Genome-wide analysis of miRNA genes in *A. thaliana* further revealed that some genomic repeats (including WGDs, tandem duplications, and segmental duplications) and following dispersal and diversification were also an essential pathway for the origin of miRNAs (Smalheiser and Torvik, 2005). Moreover, another potential source of miRNAs is random sequences and spontaneously formed from foldback sequences (Fenselau et al., 2008). As a large proportion of miRNA genes were laying within TEs or pseudogenes, the hypothesis of miRNA originating from TEs or pseudogenes has been proposed by researchers recently (Piriyapongsa and Jordan, 2008; Sasidharan and Gerstein, 2008).

Despite previously being referred to as junk DNA (Zhang et al., 2003), pseudogenes are now known to be essential elements of most eukaryotic genomes, making important contributions to their structure, diversity, capacity, and adaptation (Balasubramanian et al., 2009; Poliseno et al., 2015). The widely distributed pseudogenes are a rapidly evolving part of the genome because they have the potential for incorporating new functions into DNA sequences by mutant alleles (Balakirev and Ayala, 2003; Zhang et al., 2003). Here, when exploring the distribution of poplar miRNAs in different parts of genome regions, including the 5′ UTRs, CDS, 3′ UTRs, introns, exons, promoters, transposons, pseudogenes, and intergenic regions, we determined that pseudogenes contributed a certain proportion (14.47%) of the miRNAs. Owing to their origin as gene copies, pseudogenes typically exhibit a high sequence homology to their parent gene. Consequently, it is possible that some pseudogene-derived miRNA may be implicated in repressing transcription of their parental gene. This strong association of miRNAs with pseudogenes provides an important mechanism for the origin and posttranscriptional regulation of miRNAs (Guo et al., 2009; Xie et al., 2019).

### MicroRNA Mediated Defense Against Pathogen Stress

The plant NB-LRR genes mediate effector-triggered immunity by acting as key receptors during the innate immunity response against a wide range of pests and diseases. The NB-LRR genes are generally grouped into two subclasses: the toll/interleukin-1 receptor-like group (TIR-NB-LRRs) and a coiled-coil domain-containing group (CC-NB-LRRs) (Jones and Dangl, 2006). Both classes can be triggered by miRNAs to generate phasiRNAs, which can reduce the levels of the transcripts of their targets in *cis* and trans (Zhai et al., 2011). In contrast to low-copy genes, many NB-LRR genes have undergone dramatic duplications and losses, domain architecture variations, the partitioning of subfamilies, and copy number variation among species (Karasov et al., 2014). Thus, the NB-LRR genes are highly variable, lineage-specific, and associated with the plant immune response, providing material to allow rapid adaptative evolution.

The target sites of conserved miRNAs are often located within the highly conserved domains of the target genes (Rhoades et al., 2002). Unlike conserved miRNAs, newly evolved miRNAs tend not to target these conserved functional domains and may instead target mRNAs simply by chance (Chen and Rajewsky, 2007). Indeed, the target genes of the newly emerged miRNAs in *Populus* were found to have various functions, including numerous DR *NB-LRR* genes and few TFs. Our analysis demonstrated that more *Populus*-specific miRNAs target the NB-LRR genes than conserved miRNAs. Considering that this *de novo* diversity may be associated with plant defense, the *Populus*-specific miRNAs have a greater potential to target newly evolved plant DR genes, further contributing to the phenotypic innovation of the host. Once the newly emerged miRNAs become fixed in the regulatory modules, they may gradually evolve to target more genes linked to their specific function (Chen and Rajewsky, 2007; Xie et al., 2017).

### *Populus* MicroRNA/Target Patterns Support the Gene Dosage Balance Hypothesis

Gene duplication is one of the primary driving forces in the evolution of genomes and genetic systems (Moore and Purugganan, 2003). Duplicated genes were classified into five types, including WGD, proximal duplication, tandem duplication, transposed duplication, and dispersed duplication. As an extreme gene replication mechanism, WGD results in a sudden increase in the size of the genome and entire gene set. In contrast to small-scale duplicates, duplicates created by WGD (also called homologs) tend to be retained at much higher fractions (Rodgers-Melnick et al., 2012). Also, gene duplicability or the ability of genes to be retained following duplication is often biased. As we all know, three WGD events occurred during *Populus* evolution: an ancient duplication event, a middle event shared among the Eurosids, and a recent event shared among the Salicaceae (Tuskan et al., 2006). The modern poplar genome began to diverge around 6 million years after the “Salicoid” duplication, and retained WGD genes are biased toward more central roles in networks, such as members of signal transduction cascades and TFs (Freeling, 2009; Rodgers-Melnick et al., 2012). Therefore, genes retained as duplicate pairs following WGDs are disproportionately likely to encode TFs and components of multi-protein complexes, with a potential explanation for this phenomenon given by the gene balance hypothesis (Birchler and Veitia, 2007, 2012; Edger and Pires, 2009; Liang and Schnable, 2018).

The role of miRNAs was potentially important in terms of modulating the expression of TFs because miRNAs can operate in a dosage-sensitive manner (Guo et al., 2010). Besides, the target sites of conserved miRNAs are often located within the highly conserved domains of the target genes (Rhoades et al., 2002). Following WGDs, many of these duplicated TFs evolved separate functions in divergent ways, such as non-functionalization (Ohno, 1971), subfunctionalization (Lynch and Force, 2000) or neofunctionalization, to adapt growth/development and stress response. In this case, only one of the duplicates is targeted by miRNA, indicating a gain or loss of miRNA target site after the WGD event. Also, the evolution of miRNA binding sites suggests a coevolution between miRNAs and their targets tending to preserve core duplicates in adapting to the change of environment. Together, our study provides insights into the regulation of miRNAs and target functional evolution in the defense process.

## Data Availability Statement

The datasets generated for this study can be found in the Genome Sequence Archive in the Beijing Institute of Genomics BIG Data Center, Chinese Academy of Sciences (https://bigd.big.ac.cn/gsa) under the accession number CRA003506 (Small RNA sequencing data) and CRA001647 (RNA sequencing data).

## Author Contributions

JX designed the research. SC performed the research, analyzed the data, and wrote the manuscript. JW, YfZ, YyZ, WX, and JX revised the manuscript. YL provided valuable suggestions to the manuscript. JX obtained funding and is responsible for this article. All authors read and approved the manuscript.

## Conflict of Interest

The authors declare that the research was conducted in the absence of any commercial or financial relationships that could be construed as a potential conflict of interest.

## Footnotes

1 http://rfam.xfam.org/

2 http://www.mirbase.org/

3 https://sourceforge.net/projects/mireap/

4 http://plantgrn.noble.org/psRNATarget/

5 http://systemsbiology.cau.edu.cn/agriGOv2

6 https://sg.idtdna.com/Scitools/Applications/RealTimePCR/

## Supplementary Material

Supplementary Figure 1 Hairpin structure of 21 novel miRNAs.

Supplementary Figure 2 Validation of identified expression of miRNAs and target genes.

Supplementary Data 1 Primer sequences used for RT-qPCR.

Supplementary Data 2 Mapping results statistics.

Supplementary Data 3 Known and novel miRNAs identified in this study.

Supplementary Data 4 The expression value of each miRNA (TPM) and target genes (FPKM).

Supplementary Data 5 Conserved and Populus-specific miRNA identified in this study.

Supplementary Data 6 miRNA list in each cluster by K-means analysis.

Supplementary Data 7 The position of miRNAs in Populus genome.

Supplementary Data 8 The predicted targets of miRNAs.

Supplementary Data 9 Correlation of miRNA/target pairs (*r* < –9 × 10^–1^, *P* < 5 × 10^–2^).

Supplementary Data 10 The miRNA/target pairs having degradome evidence.

Supplementary Data 11 miRNA–TF pairs.

Supplementary Data 12 WGD pairs in TF targets.

Supplementary Data 13 WGD pairs in all target genes.

Supplementary Data 14 Transcription factor binding sites of disease-resistance related genes.
