# Supplementary figures and images for: Genome-Wide Analysis of Coding and Non-coding RNA Reveals a Conserved miR164–NAC–mRNA Regulatory Pathway for Disease Defense in Populus
Source: Front Genet. 2021 May 28;12:668940. doi: 10.3389/fgene.2021.668940 (PMC8195341; doi:10.3389/fgene.2021.668940)

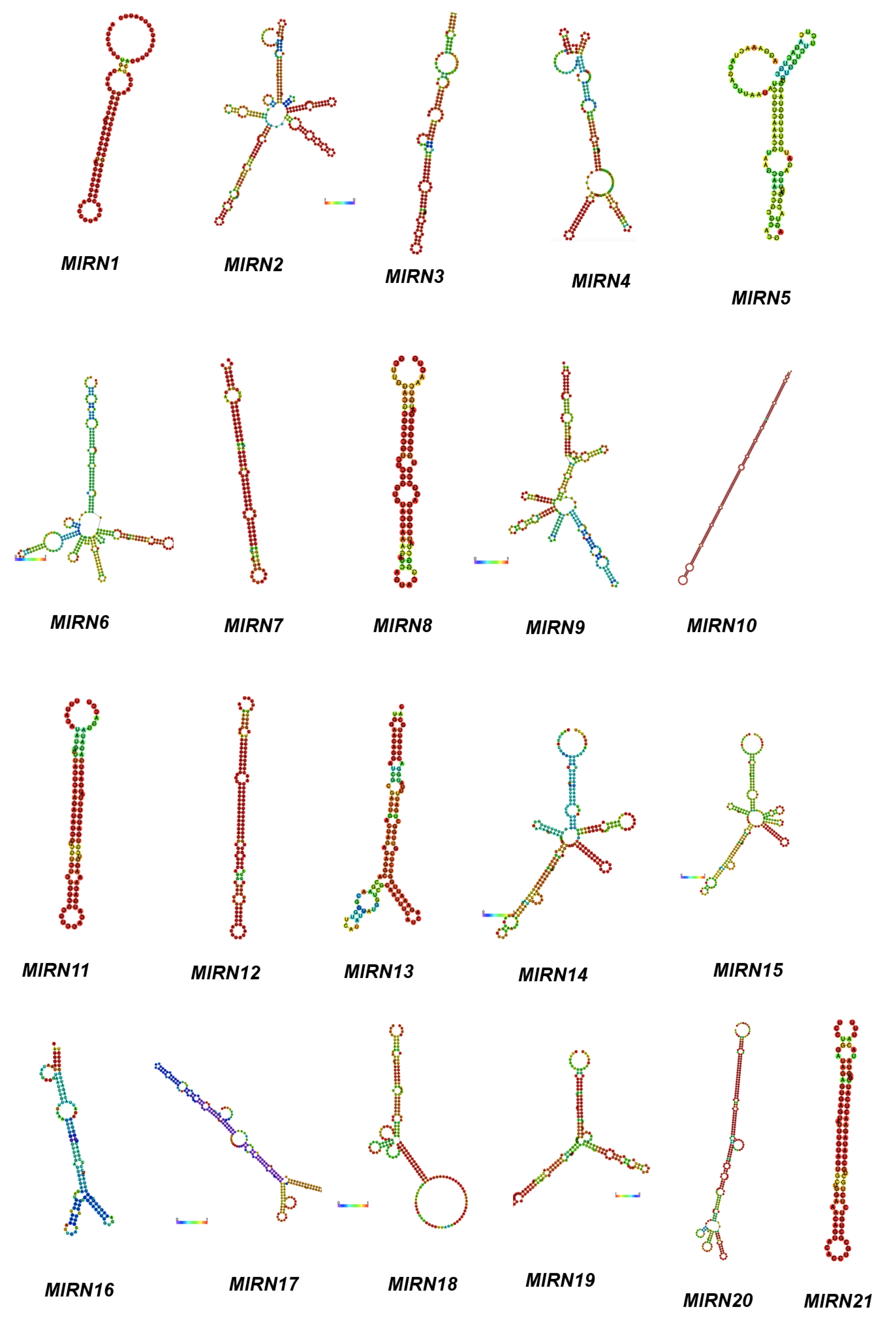

Supplement: Supplementary Figure 1 — Hairpin structure of 21 novel miRNAs. [file Image_1.TIF]

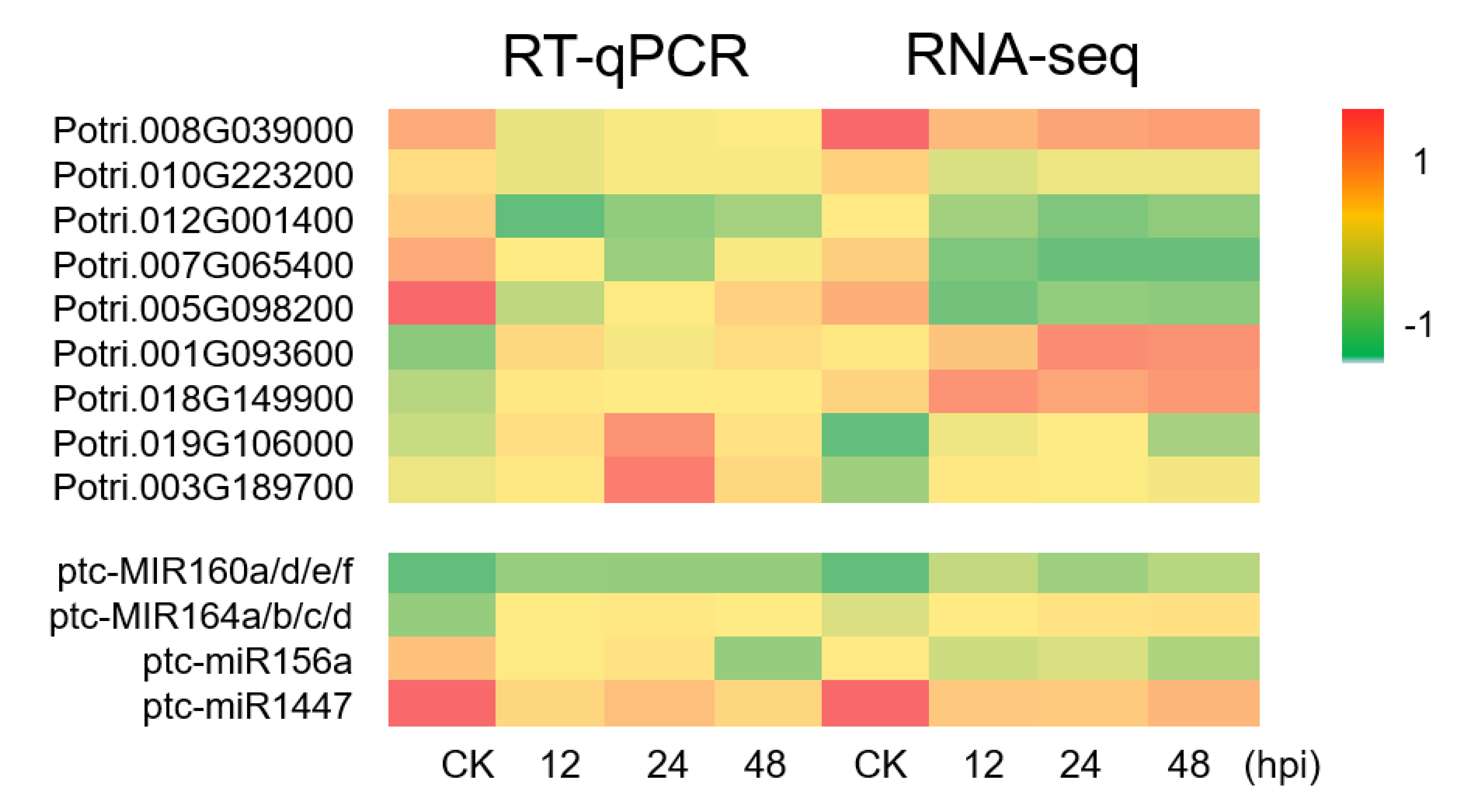

Supplement: Supplementary Figure 2 — Validation of identified expression of miRNAs and target genes. [file Image_2.TIF]
